# Analysis of mitochondrial DNA alteration in new phenotype ACOS

**DOI:** 10.1186/s12890-016-0192-6

**Published:** 2016-02-12

**Authors:** G. E. Carpagnano, D. Lacedonia, M. Malerba, G. A. Palmiotti, G. Cotugno, M. Carone, M. P. Foschino-Barbaro

**Affiliations:** Department of Medical and Surgical Sciences, Institute of Respiratory Diseases, University of Foggia, viale degli Aviatori, Foggia, 71100 Italy; Department of Internal Medicine, University of Brescia and AO Spedali Civili, Brescia, Italy; Division of Respiratory Disease, Fondazione Salvatore Maugeri, Cassano Murge, Italy

**Keywords:** Mitochondrial DNA, Asthma, COPD, ACOS, Oxidative stress

## Abstract

**Background:**

Mitochondria contain their own DNA (MtDNA) that is very sensitive to oxidative stress and as a consequence could be damaged in quantity. Oxidative stress is largely recognized to play a key role in the pathogenesis of asthma and COPD and might have a role in the new intermediate phenotype ACOS (asthma-COPD overlap syndrome). The aim of this study was to investigate MtDNA alterations, as an expression of mitochondrial dysfunction, in ACOS and to verify whether they might help in the identification of this new phenotype and in its differentiation from asthma and COPD.

**Methods:**

Ten (10) ACOS according to Spanish guidelines, 13 ACOS according to GINA guidelines, 13 COPD, 14 asthmatic patients and ten normal subjects were enrolled. They further underwent a blood, induced sputum and exhaled nitric oxide collection. Content of MtDNA and nuclear DNA (nDNA) were measured in the blood cells of patients by Real Time PCR.

**Results:**

ACOS patients showed an increase of MtDNA/nDNA ratio. Dividing ACOS according to guidelines, those from the Spanish showed a higher value of MtDNA/nDNA compared to those from GINA/GOLD (92.69 ± 7.31 vs 80.68 ± 4.16). Spanish ACOS presented MtDNA/nDNA ratio closer to COPD than asthma. MtDNA was higher in asthmatic, COPD, GINA and Spanish ACOS patients compared to healthy subjects (73.30 ± 4.47–137.0 ± 19.45–80.68 ± 4.16–92.69 ± 7.31 vs 65.97 ± 20.56).

**Conclusion:**

We found an increase of MtDNA/nDNA ratio in ACOS subjects that led us to conclude that there is presence of mitochondrial dysfunction in this disease, that makes it closer to COPD than to asthma. Although the MtDNA/nDNA ratio results are a useful marker for differential diagnosis from asthma, COPD and ACOS, further studies are needed to confirm the potentiality of MtDNA/nDNA ratio and to a better characterization of ACOS.

**Electronic supplementary material:**

The online version of this article (doi:10.1186/s12890-016-0192-6) contains supplementary material, which is available to authorized users.

## Background

In the spectrum of chronic airways diseases, asthma and chronic obstructive pulmonary disease (COPD) are the most frequent. Although they are characterized by clear differences, some manifestations of both may coexist and in this situation the clinical condition has been recently called the asthma-COPD overlap syndrome (ACOS) [1].

Today there are two international guidelines that might help in classifying ACOS: the Spanish one that defines ACOS in the presence of two major and two minor criteria such as very positive bronchodilator test, eosinophilia in sputum, history of asthma and high total IgE, personal history of atopy and positive bronchodilator test on two or more occasions [2], and the Global Initiative for Asthma (GINA) and Global Initiative for Chronic Obstructive Lung Disease (GOLD) that foresee that an ACOS patient has a similar number of features of asthma and COPD. Although a clear interest is today directed to the ACOS, because the recognition of this new phenotype might change the paradigm of asthma and COPD management from evidence based to personalized medicine [3], the main limit to this process remains that no universal definition criteria still does not exist.

More than 10.500 studies are present on pubmed on ACOS, identifying patients affected, sometimes according to Spanish and other times to GINA/GOLD guidelines. Most of them focused on clinical, functional and inflammatory differences in ACOS patients compared to asthmatic and COPD patients [4].

With this study we would like to give our contribution to the better framing of ACOS among asthma and COPD, analysing differences in ACOS classified according to Spanish and GINA/GOLD guidelines. Mitochondria are independent double membrane organelles found in the cytosol of eukaryotic cells which are involved in energy production, cell proliferation, apoptosis and intracellular calcium homeostasis [5]. The mitochondria genome is a small (16569-bp), unmethylated DNA molecule that is present in multiple copies in individual mitochondria [6]. Heteroplasmy is the coexistence of multiple mitochondrial DNA variants in a single source. Low levels of heteroplasmy have been found in almost every healthy individual who has been studied to date. MtDNA mutations that have occurred within approximately three human generations are usually heteroplasmatic. In lung disease, alterations of mitochondria have been evidenced in lung cancer [7, 8], asthma [9, 10], pneumonia, respiratory failure, recurrent aspiration, nocturnal hypoventilation, COPD [11], aging lung [12]. Recently our group reported an increased mitochondrial DNA in patients affected by obstructive sleep apnoea (OSA) [13]. We found that mtDNA/nDNA ratio in OSA patients studied positively correlated with levels of Reactive Oxygen Metabolites (ROMs) measured in paired plasma, and therefore we hypothesized that MtDNA/nDNA ratio could be considered a surrogate oxidative stress marker [13].

Determination of mitochondrial DNA content could be important for understanding many cellular processes under different diseases [12].

For example, because mitochondrial DNA is more susceptible to reactive oxygen species (ROS) induced damage compared to nuclear one, lacking protective histones and decreasing the DNA repair capacity [14], some authors suggested that it could be used as a marker of oxidative stress [15]. Since the oxidation is common in the pathogenesis of several lung diseases as obstructive lung diseases, numerous recent studies analysed mitochondrial dysfunction in asthma and COPD. Most authors agree reporting an increase and a dysfunction of mitochondrial DNA in both asthma and COPD [16–18]. However, no studies exist, to our knowledge, on mitochondrial DNA analysis in ACOS.

A convenient way to measure mitochondrial DNA content in a cell is to measure mitochondrial versus nuclear genome ratio, termed Mt/N [19]. Mt/N is attractive as a putative biomarker because it can be measured in a little as 1 pg of genomic DNA [20].

The aim of this study was to investigate presence of mitochondrial dysfunction in ACOS measuring Mt/N DNA for the first time. We aimed to verify the potentiality of MtDNA/nDNA in differential diagnosis of ACOS and give our contribution for a better definition and characterization of this new phenotype.

## Methods

### Population

Patients were recruited for the study from the out-patient facility of the Institute of Respiratory Diseases of the University of Foggia, Italy. We selected 10 ACOS according to Spanish guidelines, 13 ACOS according to GINA guidelines, 13 COPD patients, 14 asthmatic patients and ten normal subjects. Written informed consent was obtained from all subjects, and the study was approved by our institutional ethics committee of Foggia. Asthmatics were classified and treated according to GINA guidelines: we chose never–smoker patients with induced-sputum eosinophilia and with a classic reversibility of 12 % and 200 cc. COPD patients were classified according to GOLD guidelines: smoker patients with non reversible airflow limitation at spirometry and with induced-sputum neutrophilia. Furthermore, we selected Spanish ACOS patients using two major criteria: very positive bronchodilator test (increase in FEV_1_ ≥ 15 % and ≥400 mL) and eosinophilia in sputum. In addition we selected GINA ACOS patients; they should have a similar number of features for both asthma and COPD (age > 40 and neutrophilia in sputum vs. personal history of asthma and a classic reversibility) (Tables 1 and 2).

**Table 1:** Acos according to Spanish and Gina guidelines

	ACOS According to Spanish guidelines	
Major criteria	1-very positive bronchodilator test (increase in FEV1 ≥ 15 % and ≥400 mL)	
	2-Eosinophilia in sputum	
	3-Personal history of asthma	
Minor criteria	1-High levels of total IgE	
	2-Personal history of atopy	
	3-Positive bronchodilator test on at least two occasions (increase of FEV1 > 12 % and >200 mL)	
	ACOS According to GINA guidelines	
Criteria	A similar number of features of both asthma and COPD, consider the diagnosis of ACOS	
	Asthma	COPD
	1-personal history of asthma	1-age > 40
	2-Positive bronchodilator test (increase of FEV1 > 12 % and >200 mL)	2-neutrophilia in induced sputum

**Table 2:** Demographic and Clinical data of patients enrolled

	COPD		ACOS Spanish		Asthma		ACOS GINA		Healthy subjects		*p*
	mean	ds	mean	ds	mean	ds	mean	ds	mean	ds	
Age	72	4.78	67.11	6.39	60.67	12.30	61.80	6.25	61.18	3.72	0.0225
BMI	29.45	4.29	28.88	4.46	28.86	5.63	29.70	5.85	27.80	4.64	ns
Atopy (n)	0		4		10		7		0		
FEV1	46.88	10.68	72.56	23.40	88.78	17.73	83.60	22.83	90.97	6.33	0.0006
REV	6.37	2.26	18	8.48	18.89	7.63	19.60	4.81	0		0.0004
Smoking habit:											
smokers	2		0		0		2				
ex smokers	11		8		0		1				
never smokers	0		2		14		10		0		
P/Y	68.63	32.34	36.83	44.43	0	0	6.60	13			<0.0001
FENO 50	17.63	4.24	23.33	14.84	21.89	11.60	27.30	14.80	10.23	3.50	ns
induced sputum eosinophils	0.58	1.39	4.86	3.18	47.33	26.52	2.10	1.73	0.60	0.80	<0.0001
induced sputum neutrophils	88.66	10.80	82.86	10.64	24.44	20.16	81.20	11.39	27.30	13	<0.0001

*BMI:* body mass index, *FEV1:* forced expiratory volume in one 1 s, *REV:* reversibility of FEV1 after bronchodilatation test with salbutamol, *P/Y:* pack/year, *FENO 50:* Fraction of exhaled nitic oxide at 50 ml/s

All subjects enrolled were assessed during a period of stability and at least 4 weeks after an upper respiratory tract infection.

At the first visit a complete baseline questionnaire requesting information on medical history was administered to all subjects who were then given a physical examination, atopy assessment, spirometry with bronchial obstruction reversibility test. During the second visit, subjects underwent a collection of blood, exhaled nitric oxide (NO) and finally by the sputum induction.

### Atopic status

Skin prick test (SPT) was performed for a panel of inhalant allergens as previously described for common aeroallergens (Lofarma, Italy) [21].

### Lung function

Pulmonary function tests were performed. Forced expiratory volume in 1 s (FEV_1_) and forced vital capacity (FVC) were measured using a spirometer (Sensormedics, USA). The best value of three maneuvers was expressed as a percentage of the predicted normal value. After baseline evaluation, spirometry was repeated 15 min after the subjects had inhaled 400 micrograms of salbutamol as previously reported [22].

Reversibility of airways obstruction was expressed in terms of the percent changes from baseline of the forced expiratory volume in 1 s (FEV_1_).

### Induced sputum collection and processing

The sputum was induced through inhalation of hypertonic saline solution (4.5 %) with an ultrasonic nebulizer (DeVilbiss 65; DeVilbiss Corporation,Somerset, PA) in healthy subjects and in mild asthmatics and analysed after selection of plug. The collected sputum samples were examined within 2 h. Selected portions of the sputum sample were chosen through examination with an inverted microscope, and were weighed. Dithiothreitol (DTT; Sputolysin; Calbiochem Corp., San Diego, CA), freshly prepared in a 1:10 dilution with distilled water, was added in a volume (in microliters) equal to four times the weight of the selected portion (in milligrams) of each sputum specimen. The DTT–sputum mixture was placed in a shaking water bath at 37 ° C for 20 min and homogenized. It was further diluted with phosphate buffered saline (PBS) in a volume equal to the sputum plus DTT. The suspension was filtered through gauze to remove mucus, and was centrifuged at 2000 rpm for 5 min. The supernatant was aspirated and frozen at−70 °C for later analysis. The cell pellet was resuspended in a volume of PBS equal to that of the sputum plus DTT and PBS preparation described previously. Total cell count (TCC) and viability (Trypan blue exclusion method) were determined with a Burkers chamber hemocytometer. The cell suspension was placed in a Shandon 3 cytocentrifuge (Shandon Southern Instruments, Sewickley, PA) and cytospin preparations were made at 450 rpm for 6 min. Cytospin slides were fixed with methanol and were stained with May–Grunwald–Giemsa for an overall differential cell count of 500 nucleated nonsquamous cells. Only samples with a cell viability > 50 and < 20 % squamous cell contamination were considered adequate [23].

In severe asthmatic subjects we used the spontaneous sputum or the sputum produced after inhalation of isotonic saline solution. Three asthmatics, two GINA ACOS and one Spanish ACOS were not able to produce adequate sputum samples (defined as containing at least 500 non-squamous cells) and their expectorates were discharged. The sputum (spontaneous or induced) was used for cytological analysis.

### Measurement of exhaled NO

A rapid-response chemiluminescence NO analyzer (NIOX MINO, Aerocrine, Cosmed, Roma, Italy) was used to quantify oxides of nitrogen (NOs). Two-point calibrations were performed daily using 5.2-parts per million calibration gas. Exhaled NO (F_E_NO) was measured using a previously described restricted breath technique, which employed expiratory resistance and positive mouth pressure to close the velum and exclude nasal NO, and a constant expiratory flow of 50 mL/s and a 350 mL/s. Repeated exhalations were performed until three plateaus agreed within 5 % [24, 25].

### Blood collection and DNA Extraction

A blood sample was obtained in the morning. A total of 3 ml peripheral blood specimens was collected in EDTA tubes and then were stored at−80°. Whole blood DNA was extracted with QIAamp DNA MiniKit according to the manufacturer’s protocol (Qiagen, Hilden, Germany). The concentration of extracted DNA was measured at 260 nm with NanoDrop Spectrophotometer (Thermo Scientific NanoDrop, USA) and was adjusted to 10 ng/μl. Extracted DNA was stored at−20 °C until further use.

### Quantitative Real-Time PCR

Mitochondrial DNA content was measured by quantitative real time PCR method using an Applied Biosystems 7300 real-time PCR System (PE Applied Biosystems). MtDNA was measured by quantification of a unique mitochondrial fragment relative to a single copy region of beta-2-microglobulin nuclear gene (β2M). Primers, Probes (IDT, Integrated DNA Technologies, USA) and gene accession numbers are listed in Table 3. Mitochondrial DNA and β2M probes were labelled at 5’ end with 6 FAM and MAX fluorescent dyes respectively and both probes contained BHQ-1 as a quencher dye at 3’ end. The PCR mix was: 1x TaqMan® Universal PCR Master Mix (PE Applied Biosystems), 200 nM of each primer, 125 nM of TaqMan Probe, 50 ng of total genomic DNA extract in a 20 μl PCR reaction. Quantitative real-time PCR conditions were 2 min at 50 °C and 10 min at 95 °C, followed by 40 cycles of 15 s of denaturation at 95 °C and 60 s of annealing/extension at 60 °C. The data presented are the means of 3 measurements. Standard curves obtained from serial dilutions of PCR-amplified target sequences were used for the quantification of MtDNA (Mt) and nuclear genome (N) then the ratio of Mt/N DNA was calculated.

**Table 3:** Primers/probes used in the study

Gene accession number	Primer/probe	Sequence	Product size (bp)
Human mithocondrial genome NC_012920	Mito F	TTAAACACATCTCTGCCAAACC	150
	Mito R	AGATTAGTAGTATGGGAGTGGGA	
	Mito P	AA CCC TAA CAC CAG CCT AAC CAG A	
Human β2M accession number M17987	β2M F	CTTTCTGGCTGGATTGGTATCT	100
	β2M R	CAGAATAGGCTGCTGTTCCTAC	
	β2M P	AG TAG GAA GGG CTT GTT CCT GCT G	

### Analysis

Descriptive statistics (i.e., means, standard deviations, percentages) were applied to summarize the continuous and categorical variables. All variables analyzed were normally distributed and Student’s *T*-test was used to compare the mean values. Correlation between MtDNA/nDNA and clinical, functional and inflammatory data were assessed using the Spearman’s rank correlation test. P value < 0,05 was considered significant.

## Results

Among the five groups of patients we found a statistically significant difference only between the age of patients with asthma and COPD (60.67 ± 12.30 vs 72 ± 4.78, *p* < 0.05); while there were no differences regarding the age of the other groups. Furthermore we found no statistical differences in BMI among the five groups. Ten (10) asthmatic subjects, 7 GINA/GOLD ACOS and 4 Spanish ACOS resulted atopic to prick tests. Thirteen (13) were positive to dermatophagoides, 3 to dermatophagoides and tree mix, 2 to alternaria and 3 to mold. FEV1 was lower in COPD with respect to asthma (46.88 ± 10.68 vs 88.78 ± 17.73, *p* < 0.05) and with respect to GINA ACOS (46.88 ± 10.68 vs 83.60 ± 22.83) while it was similar to Spanish ACOS (46.88 ± 10.68 vs 72.56 ± 23.40); also as expected we found a significant difference between the reversibility in asthmatic patients and both ACOS groups with respect to COPD patients. FENO50 was higher in ACOS patients with respect to Asthma and COPD although there was no statistically significant difference (Additional file 1).

Considering the induced sputum neutrophilia, asthmatic patients had a statistically significant low percentage (24.44 ± 20.16, *p* < 0.05) with respect to the other groups of patients in which we found similar values instead (COPD 88.66 ± 10.80 - Spanish ACOS 82.86 ± 10.64 - GINA ACOS 81.20 ± 11.39; *p* > 0.05). While considering induced sputum eosinophilia, although we used a cut-off >4 % for identifying Spanish ACOS patients, we found a statistically significant difference only among asthmatic patients (47.33 ± 26.52, *p* < 0.05) and the other 3 groups of patients (COPD 0.58 ± 1.39 - Spanish ACOS 4.86 ± 3.18 - GINA ACOS 1.10 ± 1.73).

The MtDNA/nDNA levels in healthy non-smokers was lower compared to COPD, Spanish ACOS, GINA ACOS and asthmatic patients (65.97 ± 20.56 vs 137.0 ± 19.45, 92.69 ± 7.31, 80.68 ± 4.16 and 73.30 ± 4.47; *p* < 0.05).

**Fig. 1:**
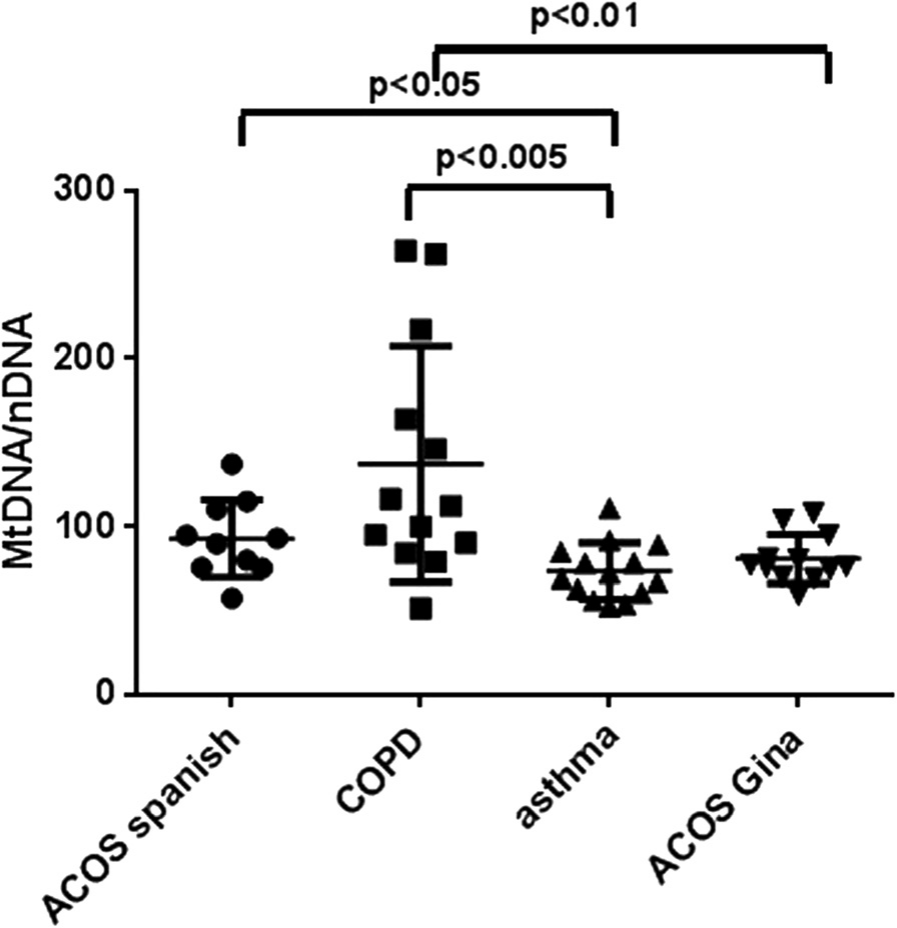
Differences between ratio Mitochondrial/nuclear DNA in ACOS according to Spanish or GINA guidelines, asthmatic and COPD patients. MtDNA/nDNA was higher in all the groups suggesting the presence of inflammation; ACOS patients present an intermediate value of MtDNA/nDNA with respect to Asthma and COPD

**Fig. 2:**
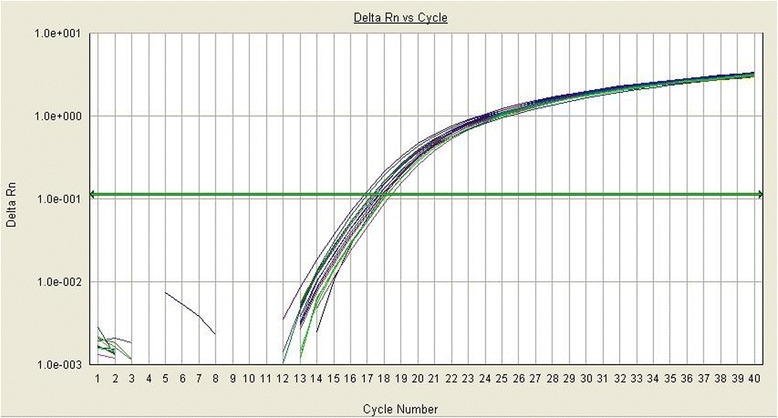
Kinetic curves of MtDNA obtained by q-Real Time PCR in whole blood from ACOS GINA patients. The reported signal (Rn) is calculated by dividing the amount of florescence emitted by the reporter by the amount of fluorescence emitted by a passive report (log ΔRn). Fluorescence is plotted vs cycle number

MtDNA/nDNA resulted higher in COPD compared to asthmatic patients (137.0 ± 19.45 vs 73.3 ± 4.47, *p* < 0.005). ACOS patients showed an increase of MtDNA/nDNA. Dividing ACOS according to guidelines, those from the Spanish showed significantly higher values of MtDNA/nDNA compared to those from GINA/GOLD (92.69 ± 7.31 vs 80.68 ± 4.16, *p* < 0.05). ACOS patients according to Spanish guidelines showed higher value of MtDNA/nDNA compared to asthmatic patients and lower compared to COPD (92.69 ± 7.31 vs 73.30 ± 4.47, *p* < 0.05; 92.69 ± 7.31 vs 137.0 ± 19.45, *p* = 0.06) (Fig. 1), while they have lower Ct compared to asthmatic and higher compared to COPD (Threshold Cycle) (0.70 ± 0.02 vs 0.72 ± 0.02, 0.70 ± 0.02 vs 0.69 ± 0.02; *p* = 0.03). ACOS patients according to GINA guidelines presented similar values of MtDNA/nDNA to asthmatic patients and significantly different from those of COPD (80.68 ± 4.16 vs 73.30 ± 4.47, *p* = ns; 80.68 ± 4.16 vs 137.0 ± 19.45, *p* < 0.01) (Fig. 1), while they have similar Ct compared to asthma and higher compared to COPD (0.72 ± 0.01 vs 0.72 ± 0.02, 0.72 ± 0.01 vs 0.69 ± 0.02; *p* = 0.03) (Fig. 2).

**Fig. 3:**
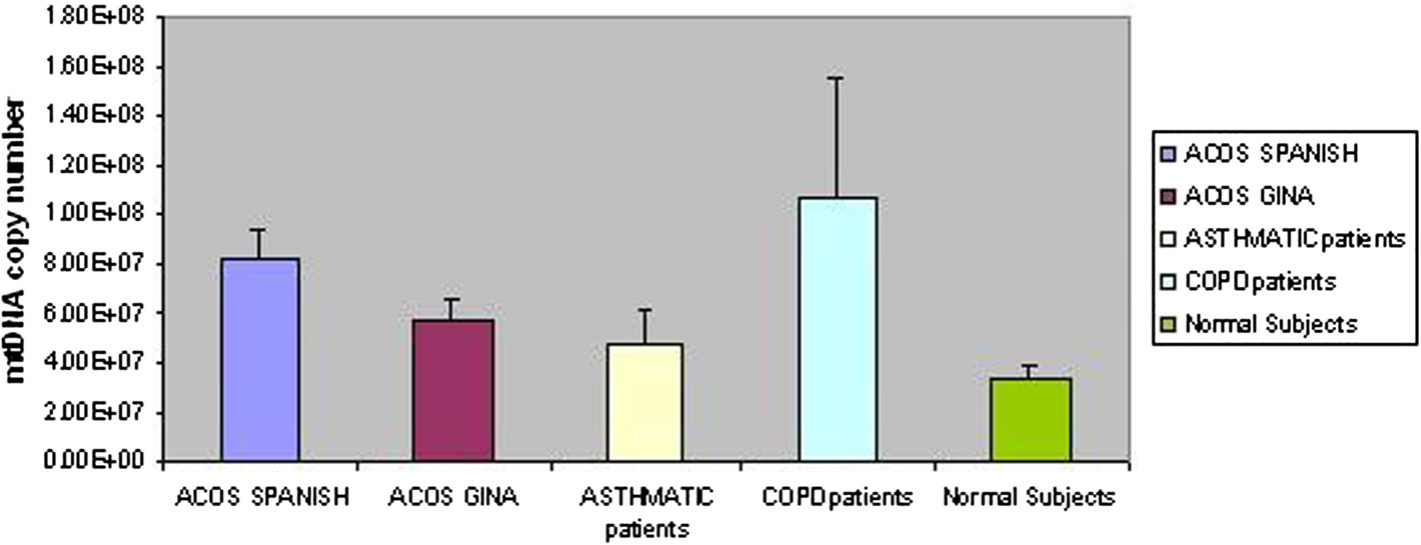
Comparison of the absolute MtDNA copy number of different groups. The values are represented with arithmetic mean and standard deviation

The MtDNA copy number data for several groups of patients and for healthy subjects were determined using the quantitative PCR (qPCR) method and were collected (Fig. 3).

**Fig. 4:**
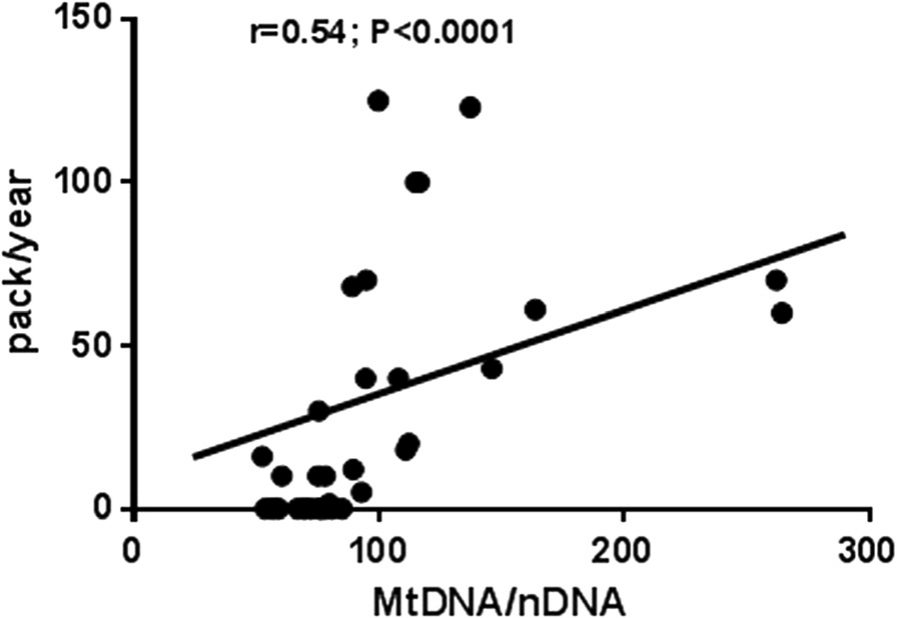
Positive correlation between ratio Mitochondrial/nuclear DNA and pack years. Cigarette smoking is associated with increased inflammation. This is further demonstrated by the increase of MtDNA/nDNA

**Fig. 5:**
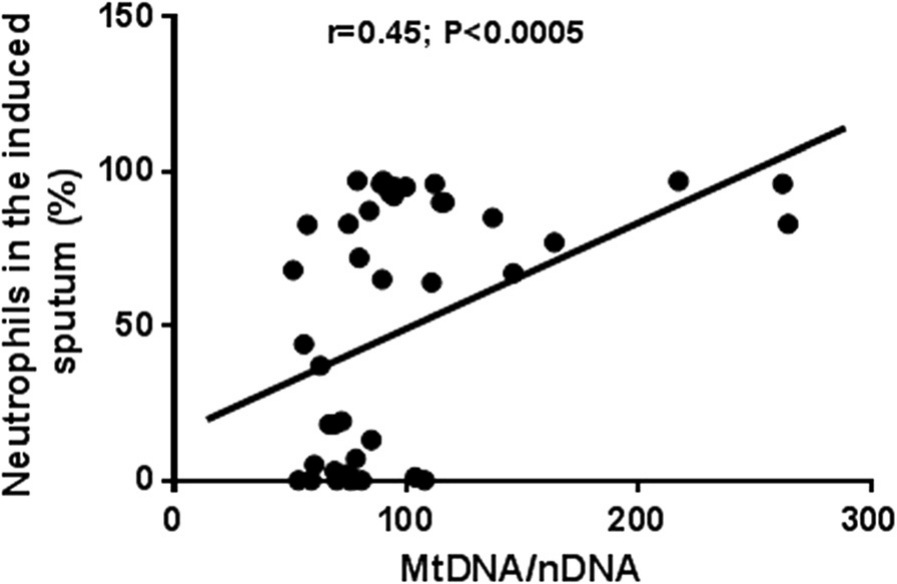
Positive correlation between ratio Mitochondrial/nuclear DNA and neutrophils percentage in the induced sputum. The presence of neutrophils in the sputum is associated with a higher grade of inflammation

The level of MtDNA/nDNA was correlated with pack/years (*r* = 0.54, *p* < 0.0001) and with percentage of neutrophils in the induced sputum (*r* = 0.45, *p* < 0.0005) (Figs. 4 and 5).

Age, body mass index (BMI), atopy, lung function, exhaled NO, eosinophils in the induced sputum did not influence mitochondrial DNA directly.

## Discussion

The main result of the study was to report, for the first time, an increase of the MtDNA/nDNA in ACOS patients. Analyzing differentially ACOS according to Spanish guidelines and those to GINA/GOLD we observed that those from GINA/GOLD were similar to the asthma group although different from COPD while those from Spanish significantly differed from asthma and were closer to COPD (Fig. 1).

The entity ACOS has recently been very discussed. Although a great interest is present for this new phenotype of COPD, mainly for the therapeutic consequences that it could have, at the moment a big confusion exists about diagnostic criteria and there is no common definition. Most international groups prefer to use Spanish guidelines for the recognition of this entity, others to use the GINA/GOLD, that however are very difficult to apply because each clinician can select different paired characteristics among asthma and COPD. The ongoing efforts in differential diagnosis of ACOS could not ignore the inflammation [26].

Analyzing singular contributions, ACOS seems to present either a systemic inflammation characterized by high sensitivity C reactive protein (PCR) and interleukin 6 in serum and an airways inflammation proved by high exhaled nitric oxide and neutrophils in the induced sputum [27, 28].

As regards the oxidative stress, no data have ever been reported although it is largely known that the oxidation is strongly linked to inflammation. In this study, starting from the hypothesis that both asthma and COPD are characterized by an increased oxidative stress, we believe that ACOS, intermediate entity among those, might have an increase of oxidative stress markers [29]. This study, born to give our contribution in a clarification of the disease, offering further data that might help in this confused moment where often ACOS is nicknamed CAOS supporting the point of view of most clinicians. Actually different schools of thought identifying ACOS sometimes according to Spanish and other times to GINA/GOLD guidelines, creating a further confusion and to add new pathogenetic information to ACOS, might help the framing of this new phenotype of obstructive lung diseases.

To better understand ACOS with its characteristics is very important because we have to remember that patients affected by the disease, present high personal and clinical costs having frequent exacerbations, important respiratory symptoms, reduced physical activity and impaired quality of life. Recently, it has been estimated that they consume from 2 to 6 fold more healthcare resources than those used by asthma and COPD patients [30–33].

As a marker of mitochondrial dysfunction we chose the MtDNA/nDNA, recently studied by our group in the blood of patients with the obstructive sleep apnea. In our previous study we found a positive correlation between MtDNA/nDNA and ROMS and suggested that this marker of mitochondrial dysfunction might also be used as an indirect marker of oxidative stress. In condition of oxidative stress, the transcriptional and replication machinery of mitochondrial biogenesis via replication of the mitochondrial genome is potentiated and the increase in mitochondrial DNA quantity resulting detectable in body fluids [34].

Previous studies analysed mitochondria in other obstructive pulmonary diseases reporting an important pathogenetic role for this microrganelle. For example Girodet et al. suggested that the mitochondrial genetic background plays a key role in asthma development [35].

Flaquer et al. highlighted the importance of the mitochondria among the factors that contribute to the risk of asthma [36].

Also regarding COPD, the oxidative DNA damage seems to be prominent in the mitochondrial genome and raises the intriguing possibility that genome and sequence-specific oxidative DNA damage could contribute to transcriptional dysregulation and cell fate decision in COPD [37]. Our study is the first analysing MtDNA/nDNA as a marker of mitochondrial dysfunction in blood of asthmatic and COPD subjects. Malik et al. previously suggested that the altered mtDNA levels, described in a wide range of human diseases, may directly contribute to pathology by causing an increased immune response resulting in chronic inflammation and oxidative stress and thus contribute to pathogenesis [34].

As expected we found a high quantity of MtDNA/nDNA that confirms the presence of an increased mitochondrial dysfunction in these diseases. The absence of a correlation between MtDNA/nDNA and positivity to skin prick tests led us to believe that it is not influenced by the atopy. For the first time we analysed mitochondrial DNA balance also in ACOS reporting an increase of MtDNA/nDNA in this new phenotype. Our findings support our hypothesis of increased mitochondrial dysfunction that might be linked to an increased oxidative stress, probably as a consequence of inflammation, in ACOS, that was expected considered its common pathogenetic mechanisms with asthma and COPD. When we analysed ACOS according to Spanish and GINA/GOLD guidelines, we observed that the entity selected according to major and minor criteria of Spanish guidelines, presented a close value of MtDNA/nDNA to COPD while that according to GINA/GOLD to asthma. Although we explored only one pathogenetic aspect of ACOS, our results leave us to support the idea that ACOS is a phenotype of COPD and might be classified according to Spanish guidelines. A further element that could come in support of our point of view on ACOS collocation is the positive correlation that we reported between MtDNA/nDNA and neutrophils. Being either COPD and ACOS according to Spanish guidelines joined by the same neutrophilic inflammation, a correlated increase also in MtDNA/nDNA further supports our hypothesis (Fig. 5).

In this study we further found a positive correlation between MtDNA/nDNA and pack/years of cigarette smoked that did not surprise us considering that cigarettes contain a high number of oxidants and furthermore it is a well-recognized cause of oxidative stress (Fig. 4).

Furthermore, cigarette smoke induces cellular senescence that is involved in the pathogenesis of COPD and probably to ACOS and leads to impaired mitophagy and perinuclear accumulation of damaged mitochondria associated with aging [38]. Previous results of the study of Aravamudan evidenced the importance of mitochondrial networks and their regulation in the context of cellular changes induced by insults such as inflammation in asthma or cigarette smoke [39].

We agree with Ahmad T et al. that stated that age could have an influence in determining MtDNA concentrations. However, the population of this study and our previous study on MtDNA were in the middle age population and didn’t allow us explore this aspect that requires further study.

The main limitation of this study is the exiguous number of subjects enrolled for each group. However this is a pilot study that requires confirmation on a larger population.

Second, there isn’t a clear consensus about this method because there is no standard value of MtDNA/nDNA available upto today, although our group previously analyzed this marker in other diseases [13].

Third, we didn’t correlate MtDNA/nDNA ratio in our population with other markers of oxidative stress such as ROMS that in our previous study help us to suggest an oxidative role of MtDNA/nDNA. We aim to overcome this shortcoming with a future study where we will focus on the oxidative characterization of MtDNA/nDNA, through the correlation of this marker with other validated markers of oxidative stress such as ROMS, 8-isoprostane, reactive oxygen species (ROS); malondialdehyde (MDA); thiobarbituric acid reacting substance (TBARS); superoxide; hydroxyl radical (OH); hydroperoxyl radical (HO_2_); peroxynitrite (ONOO^−^); hydrogen peroxide (H_2_O_2_); nitrogen dioxide (NO_2_); myeloperoxidase (MPO); Oxidized low-density lipoprotein OxLDL; reduced glutathione (GSH).

Finally, we analyzed the MtDNA/nDNA in blood and not in airways of subjects enrolled. Because the inflammation and the oxidative stress in asthma and COPD was reported as either systemic or local, to compare MtDNA/nDNA in blood and airways might be important for a more clear understanding of pathogenetic mechanisms under these diseases and particularly under ACOS. To overcome this limit we are planning a further study where we will investigate this aspect.

## Conclusion

In conclusion, we reported for the first time an increase of MtDNA/nDNA in ACOS subjects as mitochondrial dysfunction. Confirmation of the correlation with other oxidative stress markers as ROMS could be considered an indirect expression of an elevated oxidative stress, and supported clinical potentiality of this new marker. Since this increase in ACOS patients selected according to Spanish guidelines is close to COPD, we support the idea that ACOS could be a phenotype of COPD more than a new different entity among asthma and COPD. In consideration of the need-to better characterize ACOS we evidence the necessity of future clinical and research contributions in this topic like this study to improve our understanding of this disease and to achieve the best management strategies [4].

### Ethics, consent and permissions

Written informed consent was obtained from all subjects, and the study was approved by the our institutional ethics committee of Foggia.

## Additional file

Additional file 1:
**Results: Antrophometric and clinical differences among groups.** (DOCX 12 kb)

## Abbreviations

MtDNA: mitochondrial DNA
nDNA: nuclear DNA
MtDNA/nDNA: mitochondrial DNA/nuclear DNA
COPD: Chronic obstructive pulmonary disease
ACOS: Asthma-copd overlap syndrome
GINA: Global initiative for asthma
GOLD: Global initiative for chronic obstructive lung disease
ROS: Reactive oxygen species
SPT: Skin prick test
FeNO: Exhaled nitric oxide
