# Supplementary material for: Analysis of mitochondrial DNA alteration in new phenotype ACOS
Source: BMC Pulm Med. 2016 Feb 12;16:31. doi: 10.1186/s12890-016-0192-6 (PMC4751730; doi:10.1186/s12890-016-0192-6)
Supplement: Additional file 1: — Results: Antrophometric and clinical differences among groups. (DOCX 12 kb) [file 12890_2016_192_MOESM1_ESM.docx]

**Results**

**Antrophometric and clinical differences among groups**

We found a statistically significant difference in age among patients with asthma and COPD (60.67±12.3 vs 72±4.781 p<0.05) while there were no differences in others groups.

We didn’t find no statistically differences in BMI among the 4 groups (COPD29,45±4.29 ; ACOS SPANISH 28.88 ±4.463; ASTHMA 28.86±5.639; ACOS GINA 29.7± 5.856; P= ns).

FEV_1_ was lower in COPD respect to asthma (46.88±10.68 vs 88.78±17.73 p<0.05) and respect to ACOS according GINA (46.88±10.68 vs 83.6±22.83) while was similar to ACOS according Spanish guidelines.

We found a significant difference between the reversibility test in asthmatic patients, and both ACOS groups respect to COPD patients (FEV_1_ post salbutamol: 18.89±7.639; 18±8.485; 19,6 ±4.812 vs 6.375±2.264).

FENO50 was higher in ACOS patients respect to Asthma and COPD although there were no a statistically significant differences (COPD 17.63±4.241; ACOS SPANISH 23.33±14.84; ASTHMA 21.89±11.6; ACOS GINA 27.3±14.8).

Asthmatic patients showed a statistically significant low percentage of neutrophils in the induced sputum (24.44±20.16 % p<0.05) respect to the others groups (COPD 88.66 ±10.8% - ACOS Spanish 82.86±10.64% - ACOS GINA 81.2±11.39%). As regard the percentage of eosinophils in the induced sputum although we used a cut-off >4% for identifying ACOS according Spanish guidelines patients, we found a statistically significant difference only among asthmatic patients (47.33±26.52 %, p<0,05) and the others 3 groups (COPD 0.58±1.39 - ACOS Spanish 4.86±3.18 - ACOS GINA 1.1±1.73 (table 3).
